# Gray Matter Thickness and Subcortical Nuclear Volume in Men After SARS-CoV-2 Omicron Infection

**DOI:** 10.1001/jamanetworkopen.2023.45626

**Published:** 2023-11-30

**Authors:** Yanyao Du, Wei Zhao, Sihong Huang, Chuxin Huang, Chang Li, Yanjing Chen, Yijie Huang, Longtao Yang, Cong Li, Huiting Zhang, Hu Guo, Jun Liu

**Affiliations:** 1 Department of Radiology, Second Xiangya Hospital of Central South University, Changsha, China; 2 Clinical Research Center for Medical Imaging in Hunan Province, Changsha, China; 3 Department of Radiology Quality Control Center, Hunan Province, Changsha, China; 4 MR Scientific Marketing, Siemens Healthineers Ltd, Wuhan, China; 5 MR Application, Siemens Healthineers Ltd, Changsha, China

## Abstract

**Importance:**

The clinical manifestations and effects on the brain of the SARS-CoV-2 Omicron variant in the acute postinfection phase remain unclear.

**Objective:**

To investigate the pathophysiological mechanisms underlying clinical symptoms and changes to gray matter and subcortical nuclei among male patients after Omicron infection and to provide an imaging basis for early detection and intervention.

**Design, Setting, and Participants:**

In this cohort study, a total of 207 men underwent health screening magnetic resonance imaging scans between August 28 and September 18, 2022; among them, 98 provided complete imaging and neuropsychiatric data. Sixty-one participants with Omicron infection were reevaluated after infection (January 6 to 14, 2023). Neuropsychiatric data, clinical symptoms, and magnetic resonance imaging data were collected in the acute post-Omicron period, and their clinical symptoms were followed up after 3 months. Gray matter indexes and subcortical nuclear volumes were analyzed. Associations between changes in gray matter and neuropsychiatric data were evaluated with correlation analyses.

**Exposures:**

Gray matter thickness and subcortical nuclear volume change data were compared before and after Omicron infection.

**Main Outcomes and Measures:**

The gray matter indexes and subcutaneous nuclear volume were generated from the 3-dimensional magnetization-prepared rapid acquisition gradient echo and were calculated with imaging software.

**Results:**

Ninety-eight men underwent complete baseline data collection; of these, 61 (mean [SD] age, 43.1 [9.9] years) voluntarily enrolled in post-Omicron follow-up and 17 (mean [SD] age, 43.5 [10.0] years) voluntarily enrolled in 3-month follow-up. Compared with pre-Omicron measures, Beck Anxiety Inventory scores were significantly increased (median, 4.50 [IQR, 1.00-7.00] to 4.00 [IQR, 2.00-9.75]; *P* = .006) and depressive distress scores were significantly decreased (median, 18.00 [IQR, 16.00-20.22] to 16.00 [IQR, 15.00-19.00]; *P* = .003) at the acute post-Omicron follow-up. Fever, headache, fatigue, myalgia, cough, and dyspnea were the main symptoms during the post-Omicron follow-up; among the participants in the 3-month follow-up, fever (11 [64.7%] vs 2 [11.8%]; *P* = .01), myalgia (10 [58.8%] vs 3 (17.6%]; *P* = .04), and cough (12 [70.6%] vs 4 [23.5%]; *P* = .02) were significantly improved. The gray matter thickness in the left precuneus (mean [SD], 2.7 [0.3] to 2.6 [0.2] mm; *P* < .001) and right lateral occipital region (mean [SD], 2.8 [0.2] to 2.7 [0.2] and 2.5 [0.2] to 2.5 [0.2] mm; *P* < .001 for both) and the ratio of the right hippocampus volume to the total intracranial volume (mean [SD]. 0.003 [0.0003] to 0.003 [0.0002]; *P* = .04) were significantly reduced in the post-Omicron follow-up. The febrile group had reduced sulcus depth of the right inferior parietal region compared with the nonfebrile group (mean [SD], 3.9 [2.3] to 4.8 [1.1]; *P* = .048. In the post-Omicron period, the thickness of the left precuneus was negatively correlated with the Beck Anxiety Inventory scores (*r* = −0.39; *P* = .002; false discovery rate *P* = .02), and the ratio of the right hippocampus to the total intracranial volume was positively correlated with the Word Fluency Test scores (*r* = 0.34; *P* = .007).

**Conclusions and Relevance:**

In this cohort study of male patients infected with the Omicron variant, the duration of symptoms in multiple systems after infection was short. Changes in gray matter thickness and subcortical nuclear volume injury were observed in the post-Omicron period. These findings provide new insights into the emotional and cognitive mechanisms of an Omicron infection, demonstrate its association with alterations to the nervous system, and verify an imaging basis for early detection and intervention of neurological sequelae.

## Key Points

**Question:**

What are the clinical manifestations and brain microstructural changes associated with the SARS-CoV-2 Omicron variant in the acute phase after infection?

**Findings:**

In this cohort study of 61 male patients with Omicron infection, the gray matter thickness in the left precuneus and right lateral occipital region and the ratio of the right hippocampus volume to the total intracranial volume were significantly reduced in the acute phase. Gray matter thickness and subcortical nuclear volume injury were significantly associated with anxiety and cognitive function.

**Meaning:**

These findings may provide new insights into the emotional and cognitive mechanisms affected by an Omicron infection, demonstrate its association with nervous system symptoms, and provide an imaging basis for early detection and intervention for neurological sequelae.

## Teaser

This cohort study explores clinical symptoms and changes to gray matter thickness and subcortical nuclear volume in the acute and 3-month follow-up phases of SARS-CoV-2 Omicron infection among adult male patients.

## Introduction

According to a report by the Global Initiative on Sharing Avian Influenza Data,^1^ genomic surveillance and preliminary phylogenetic analysis of data from China since the abolition of the zero COVID-19 policy (in December 2022) have shown that the patterns of introduced variation and risks in China were similar to those seen globally, and the strains infecting China were dominated by the SARS-CoV-2 Omicron variant. During the Omicron variant phase of the pandemic in China, from December 1, 2022, through March 1, 2023, we collected alveolar lavage fluid from 157 hospitalized patients, of whom 40 were diagnosed with COVID-19. Subsequently, 6 cases underwent genetic sequencing of the Omicron variant, and the results were Omicron BA.5.2. Several follow-up studies^2,3,4^ have shown that, similar to infections of the SARS-CoV-2 Delta variant, Omicron infection has multiple systemic symptoms, including respiratory, neurological, and digestive symptoms, and neurological sequelae are becoming the focus of attention.

Long-term COVID-19 neurological sequelae (≥90 days) are diverse and include clinical symptoms (dizziness, headache, olfactory and taste disturbances, and motor delay),^3,4,5,6^ neuropsychiatric symptoms (insomnia, depression, anxiety, and reduced cognitive function),^6,7,8^ and structural and functional changes in the brain.^3,4,7,9,10^ In addition, severe neurological symptoms such as acute ischemic stroke, encephalitis, and acute necrotizing encephalopathy in the acute phase of SARS-CoV-2 infection (14-29 days) in individual cases have also been reported in the literature.^11,12,13^ However, few individuals with mild neurological symptoms in the acute phase receive comprehensive neuropsychiatric assessment and magnetic resonance imaging (MRI) examinations, and few studies have focused on neuropsychiatric changes and brain microstructural damage after infection with the Omicron variant. Therefore, we aimed to explore clinical symptoms and brain structural changes in the acute phase of SARS-CoV-2 Omicron infection (hereinafter referred to as the post-Omicron phase) for early detection of and intervention for possible neurological sequelae to alleviate the burden on society and the health care system.

We prospectively collected clinical symptom data, neuropsychiatric findings, and MRI examination results of the same group of participants, with the aim of exploring the changes in brain structure between the pre-Omicron and post-Omicron phases and the changes in symptoms across systems in the post-Omicron phase and 3 months after infection. The changes in gray matter and subcortical nucleus volume were investigated in a data-driven manner. Finally, correlation analysis was performed to explore whether brain microstructure alterations were associated with neuropsychiatric scale scores.

## Methods

### Participants and Study Design

This prospective cohort study was approved by the Ethics Committee of the Second Xiangya Hospital of Central South University, Hunan, China, and all participants gave their written informed consent. This study followed the Strengthening the Reporting of Observational Studies in Epidemiology (STROBE) reporting guidelines for cohort studies.

**Figure 1.**
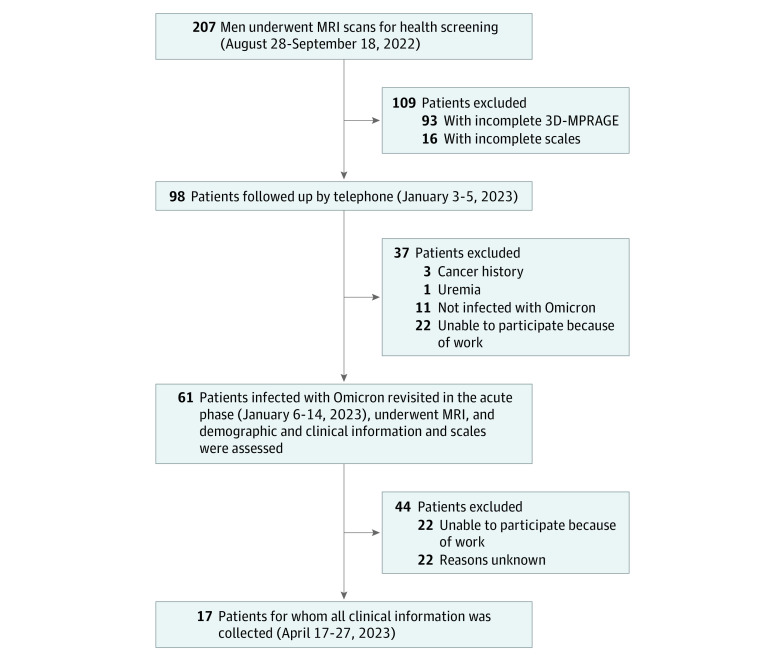
Flowchart of the Study 3D-MPRAGE indicates 3-dimensional magnetization-prepared rapid acquisition gradient echo; MRI, magnetic resonance imaging.

The experimental design is shown in Figure 1. The inclusion criteria were as follows: (1) a certain frequency of nucleic acid tests was maintained and none of the test results were positive; (2) no typical symptoms of SARS-CoV-2 infection; (3) a self-reported first infection of the virus in December 2022 and a positive nucleic acid test result; and (4) a complete set of first and second MRI and neuropsychiatric scale data. The exclusion criteria were as follows: (1) a negative nucleic acid test result; (2) an MRI contraindication; (3) a history of structural brain abnormalities, such as intracerebral hemorrhage, encephalitis, epilepsy, and psychiatric illness; and (4) a history of tumor or endocrine diseases. Ultimately, 61 individuals were included in our study. Seventeen individuals completed follow-up of clinical symptoms 3 months after infection via a web-based questionnaire. Pre-Omicron data collection occurred from August 28 to September 18, 2022; post-Omicron follow-up data collection, January 6 to 14, 2023; and 3-month follow-up data collection, April 17 to 27, 2023. All participants were Chinese Han ethnicity.

When evaluating clinical symptoms, we defined an axillary temperature above 37.5 °C as fever according to the traditional definition of fever. Based on previous studies that found significant neurological effects of axillary temperatures above 38.5 °C,^14^ we used the axillary temperature of 38.5 °C as the boundary to divide participants into febrile and nonfebrile groups and to explore structural changes.

After the first and second MRI examinations, the patients completed questionnaires, including the Beck Depression Inventory, Beck Anxiety Inventory (BAI), Insomnia Severity Index, and Regulatory Emotional Self-Efficacy scale (RESE). Cognitive tests and clinical symptom data collection were completed only after the second MRI examination. Three different domains of emotion regulation were assessed with the RESE: positive emotions, anger-irritation, and despondency-distress (DES). The cognitive tests included (1) Logical Memory, with subtests A and B performed immediately and repeated 30 minutes later; (2) the Digit Symbol Substitution Test, which assessed processing speed, sustained attention, and working memory; (3) the Knowledge subscale of the Wechsler Intelligence Scale, which measured breadth of knowledge, learning, acceptance, and the ability to understand aspects of daily life; (4) the Digit Span Task, which assessed visual and visuospatial sequence representation with forward digit span and backward digit span, respectively; and (5) the Word Fluency Test (WFT), in which the participants were asked to name as many animals as possible in 1 minute. Clinical symptoms in the acute phase were compared with those at the 3-month follow-up, and brain gray matter and subcortical nuclear volume changes were compared between the pre-Omicron and post-Omicron groups and the febrile and nonfebrile groups.

### MRI Acquisition Parameters

We acquired MRI data using one of two 3T scanners (MAGETOM Skyra [Siemens Healthcare] and uMR 790 [United Imaging Healthcare]) with 32-channel head coils. To minimize motion artifacts, participants were kept in a supine position while wearing earplugs, and a foam pad was placed between their head and the coil. The MRI scanning sequences included T1-weighted imaging, T2-weighted imaging, fluid-attenuated inversion recovery imaging, and 3-dimensional magnetization-prepared rapid acquisition gradient echo (3D-MPRAGE). The 3D-MPRAGE scanning parameters for the MAGETOM Skyra scanner were as follows: 176 sagittal sections, repetition time of 2000 milliseconds, echo time of 2.26 milliseconds, flip angle of 8°, voxel size of 1 × 1 × 1 mm^3^, section thickness of 1 mm, and field of view of 256 × 256 mm^2^. The 3D-MPRAGE scanning parameters for the uMR 790 scanner were as follows: 160 sagittal sections, repetition time of 2000 milliseconds, echo time of 3.1 milliseconds, flip angle of 10°, voxel size of 1 × 1 × 1 mm^3^, section thickness of 1 mm, and field of view of 256 × 256 mm^2^.

### MRI Data Preprocessing

The gray matter indexes and subcutaneous nuclear volume were calculated with FreeSurfer, version 6.0.^15^ The preprocessing steps included checking image quality; motion correction; removing the neck, scalp, and skull; intensity normalization; Talairach registration; segmentation of subcortical white matter and subcortical nuclei; gray matter and white matter boundary delineation; topology correction; surface deformation; registering each person’s brain to a common spherical atlas; manual correction for inaccurate cortical segmentation of the image; and calculation of the thickness, curvature, sulcus depth, and subcortical nuclear volume.^16,17^

### Statistical Analysis

#### Main Analysis

Clinical information, neuropsychiatric scale data, and the ratio of 12 subcortical nuclei volumes to the total intracranial volume (TIV) were assessed with SPSS Statistics, version 24.0 (IBM Corporation). Quantitative data were expressed as the mean (SD) or median (IQR), and categorical data were expressed as numbers and percentages. Paired-sample and 2-sample Wilcoxon-Mann-Whitney tests, 2-sample *t* tests, χ^2^ tests, and McNemar tests were used for various comparisons. In the cross-sectional MRI data comparison, general linear modeling was performed, and age and history of nicotine use were included as covariates. Monte Carlo simulation cluster analysis was used to correct for multiple comparisons.^15^ Two-sided *P* < .05 indicated statistical significance.

#### Post Hoc Region-of-Interest Analysis

The gray matter indexes were extracted from each participant and displayed in a violin plot. Pearson correlation or Spearman correlation was used depending on whether the variables were normally distributed. All correlation analysis results were corrected by the false discovery rate (FDR) for multiple comparisons.

## Results

### Participant Characteristics and Clinical Symptoms

A summary of patient characteristics is shown in Table 1. This study included 61 men with a mean (SD) age of 43.1 (9.9) years (range, 26-60 years), and the mean (SD) interval between infection and MRI scans was 21.6 (5.2) days (range, 6-34 days). Twenty-nine participants received 1 or 2 doses of the vaccine, 17 received 3 doses, and 15 did not report their vaccination status. Compared with pre-Omicron measurements, BAI scores were significantly increased (median, 4.50 [IQR, 1.00-7.00] to 4.00 [IQR, 2.00-9.75]; *P* = .006) and DES scores were significantly decreased (median, 18.00 [IQR, 16.00-20.22] to 16.00 [IQR, 15.00-19.00]; *P* = .003) at the post-Omicron follow-up (Table 1). Age (mean [SD], 40.4 [9.8] vs 47.5 [8.3] years; *P* = .006) and history of nicotine use (16 of 36 [44.4%] vs 18 of 25 [72.0%]; *P* = .03) showed significant differences between the febrile and nonfebrile groups, respectively (eTable 1 in Supplement 1), but there were no significant differences in the other characteristics or the neuropsychiatric and neurocognitive test scales.

**Table 1.** Summary of Patient Characteristics^a^

Characteristic	Pre-Omicron period (n = 61)	Post-Omicron follow-up (n = 61)	*z* Score^b^	*P* value
Age, mean (SD) [range], y	43.05 (9.87) [26-60]	NA	NA	NA
No. men/women	61/0	NA	NA	NA
Educational level, mean (SD) [range], y	15.6 (1.2) [12-20]	NA	NA	NA
BMI, mean (SD) [range]	26.2 (4.0) [21.1-42.3]	NA	NA	NA
Nicotine use, No. (%)	34 (55.7)	NA	NA	NA
Alcohol use, No. (%)	36 (59.0)	NA	NA	NA
Hypertension, No. (%)	9 (14.8)	NA	NA	NA
Type 2 diabetes, No. (%)	6 (9.8)	NA	NA	NA
Handedness, No. right/left	61/0	NA	NA	NA
Interval between Omicron infection and MRI examination, mean (SD) [range], d	21.6 (5.2) [6-34]	NA	NA	NA
Vaccination status, No. of participants				
Single or double vaccinated	29	NA	NA	NA
Booster vaccinated	17	NA	NA	NA
Missing	15	NA	NA	NA
Psychometric tests, median (IQR)				
BDI	1.00 (0.00-2.00)	1.00 (0.00-2.00)	−0.191	.849
BAI	4.50 (1.00-7.00)	4.00 (2.00-9.75)	−2.764	.006^b^
ISI	6.00 (2.00-7.00)	4.00 (1.00-7.00)	−0.644	.519
RESE, median (IQR) score				
Expressing positive emotions	13.00 (11.00-15.00)	13.00 (12.00-15.00)	−1.463	.144
Managing anger-irritation	16.00 (12.00-19.00)	15.00 (12.00-18.00)	−1.101	.271
Managing despondency-distress	18.00 (16.00-20.00)	16.00 (15.00-19.00)	−2.931	.003^b^
Neurocognitive tests, median (IQR) score				
Task A	NA	10.00 (8.00-13.00)	NA	NA
Task B	NA	8.00 (5.25-10.00)	NA	NA
DSST	NA	78.00 (65.00-93.00)	NA	NA
Knowledge subscale of Wechsler Intelligence scale, median (IQR) score	NA	24.00 (21.00-26.00)	NA	NA
FDS	NA	14.00 (12.00-14.00)	NA	NA
BDS	NA	8.00 (6.00-12.75)	NA	NA
WFT	NA	21.00 (16.00-26.75)	NA	NA

Abbreviations: BAI, Beck Anxiety Inventory; BDI, Beck Depression Inventory; BDS, backward digit span; BMI, body mass index (calculated as weight in kilograms divided by height in meters squared); DSST, Digital Symbol Substitution Test; FDS, forward digit span; ISI, Insomnia Severity Index; MRI, magnetic resonance imaging; NA, not applicable; RESE, Regulatory Emotional Self-Efficacy scale; WFT, Word Fluency Test.

^a^ Pre-Omicron indicates before infection with Omicron; post-Omicron, acute phase of follow-up (14-29 days).

^b^ Calculated using paired-samples Wilcoxon-Mann-Whitney test.

Data from 61 participants with neurological, respiratory, and digestive symptoms were analyzed in the post-Omicron follow-up. Among the neurological symptoms, fever had the highest incidence (49 [80.3%]) but the shortest duration (mean [SD], 1.4 [0.6] days); slowed reaction speed had the longest duration (8.9 [7.8] days). Among the respiratory symptoms, cough had the highest incidence (36 [59.0%]), lasting for a mean (SD) duration of 8.1 (7.2) days, and dyspnea had the longest duration (mean [SD], 11.0 [9.5] days). Among the digestive symptoms, decreased appetite had the highest incidence (22 participants [36.1%]) and the longest duration (mean [SD], 6.4 [5.8] days). Compared with the post-Omicron follow-up, the 17 individuals in the 3-month follow-up showed significant improvements in fever (11 participants [64.7%] vs 2 [11.8%]; *P* = .01), myalgia (10 [58.8%] vs 3 (17.6%]; *P* = .04), and cough (12 [70.6%] vs 4 [23.5%]; *P* = .02) (Table 2 and the eFigure in Supplement 1).

**Table 2.** Clinical Information in the Acute Phase and 3-Month Follow-Up Assessments for Omicron Infection

Symptom	Acute phase		No. (%)		*P* value^a^
	No. (%) of participants (n = 61)	Duration, mean (SD), d	Acute phase (n = 17)	3-mo Follow-up (n = 17)	
Fever^b^	49 (80.3)	1.4 (0.6)	11 (64.7)	2 (11.8)	.01
Headache	25 (41.0)	3.1 (2.3)	7 (41.2)	6 (35.3)	>.99
Fatigue	41 (67.2)	6.8 (6.2)	7 (41.2)	8 (47.1)	>.99
Myalgia	36 (59.0)	4.0 (4.3)	10 (58.8)	3 (17.6)	.04
Olfactory loss	14 (23.0)	7.3 (5.3)	3 (17.6)	2 (11.8)	>.99
Taste loss	20 (32.8)	6.1 (3.2)	1 (5.9)	0	>.99
Slower reaction time	16 (26.2)	8.9 (7.8)	5 (29.4)	2 (11.8)	.25
Motor delay	18 (29.5)	7.9 (6.1)	5 (29.4)	4 (23.5)	>.99
Cough	36 (59.0)	8.1 (7.2)	12 (70.6)	4 (23.5)	.02
Expectoration	26 (42.6)	6.3 (4.2)	7 (41.2)	6 (35.3)	>.99
Dyspnea	5 (8.2)	11.0 (9.5)	1 (5.9)	5 (29.4)	.13
Chest tightness	10 (16.4)	6.6 (4.2)	4 (23.5)	2 (11.8)	.63
Chest pain	6 (9.8)	4.5 (2.2)	1 (5.9)	1 (5.9)	>.99
Decreased appetite	22 (36.1)	6.4 (5.8)	8 (47.1)	4 (23.5)	.22
Nausea	8 (13.1)	2.5 (1.9)	4 (23.5)	2 (11.8)	.63
Vomiting	4 (6.6)	1.0 (0.0)	2 (11.8)	0	.47
Diarrhea	12 (19.7)	3.3 (1.9)	3 (17.6)	2 (11.8)	>.99

^a^ Calculated using the McNemar test.

^b^ Defined as an axillary temperature above 37.5 °C.

### Gray Matter and Subcortical Nuclear Volume Analysis

**Figure 2.**
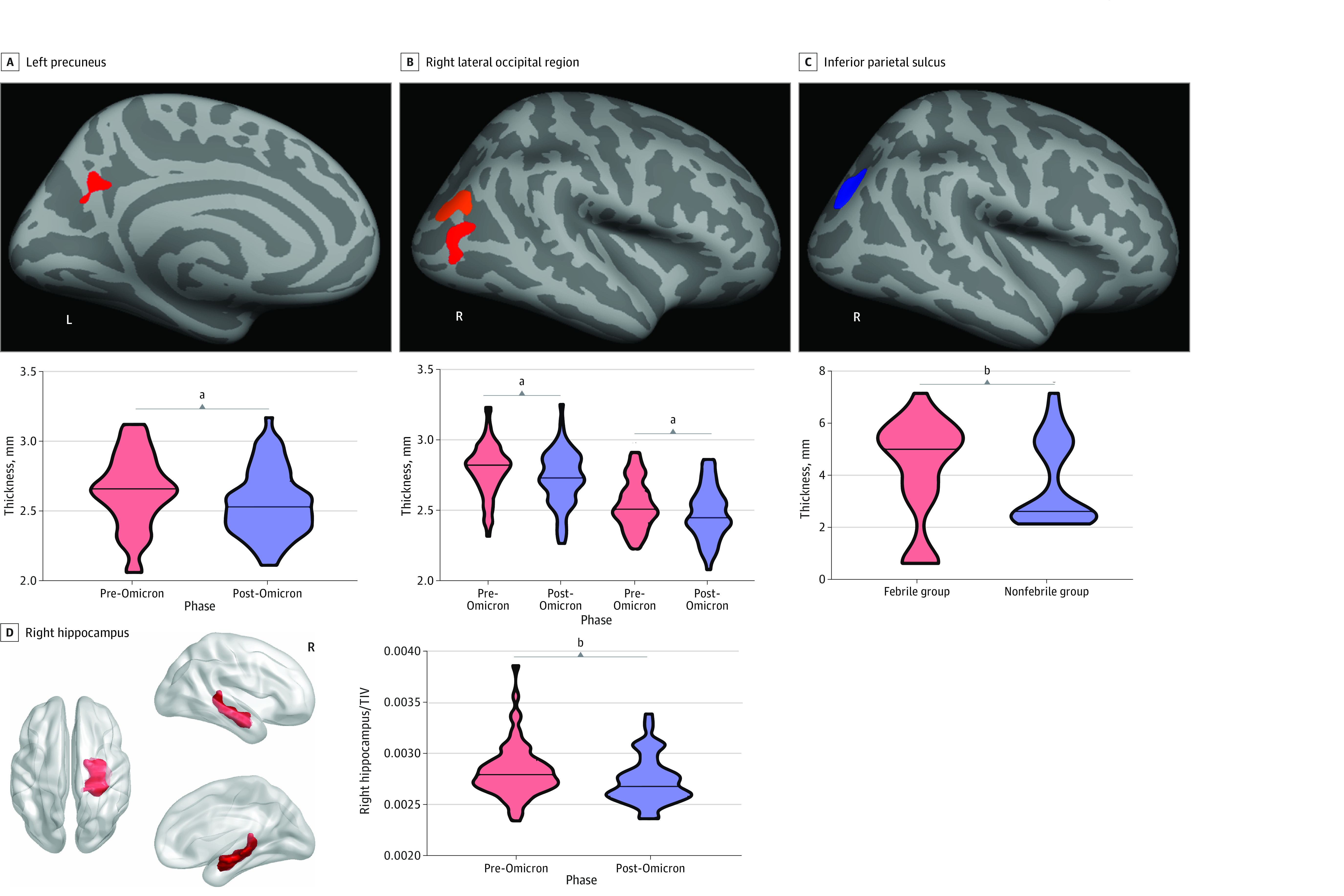
Results of Gray Matter and Subcortical Nuclear Volume Analysis and Post Hoc Region of Interest Analysis Comparing Pre-Omicron and Post-Omicron Evaluations and the Febrile and Nonfebrile Groups The pre-Omicron measurements were obtained before Omicron infection; the post-Omicron measurements, during the acute phase of follow-up (6-34 days). A, Compared with the pre-Omicron thickness, the post-Omicron thickness was significantly reduced in the left precuneus (red). B, Compared with the pre-Omicron thickness, the post-Omicron thickness was significantly reduced in the right lateral occipital region (red and orange). C, The febrile group had a significantly reduced inferior parietal sulcus depth compared with the nonfebrile group (blue). D, Compared with the pre-Omicron period, the ratio of the right hippocampus volume to the total intracranial volume (TIV) showed a significant reduction in the post-Omicron period. L indicates left; R, right.

**Figure 3.**
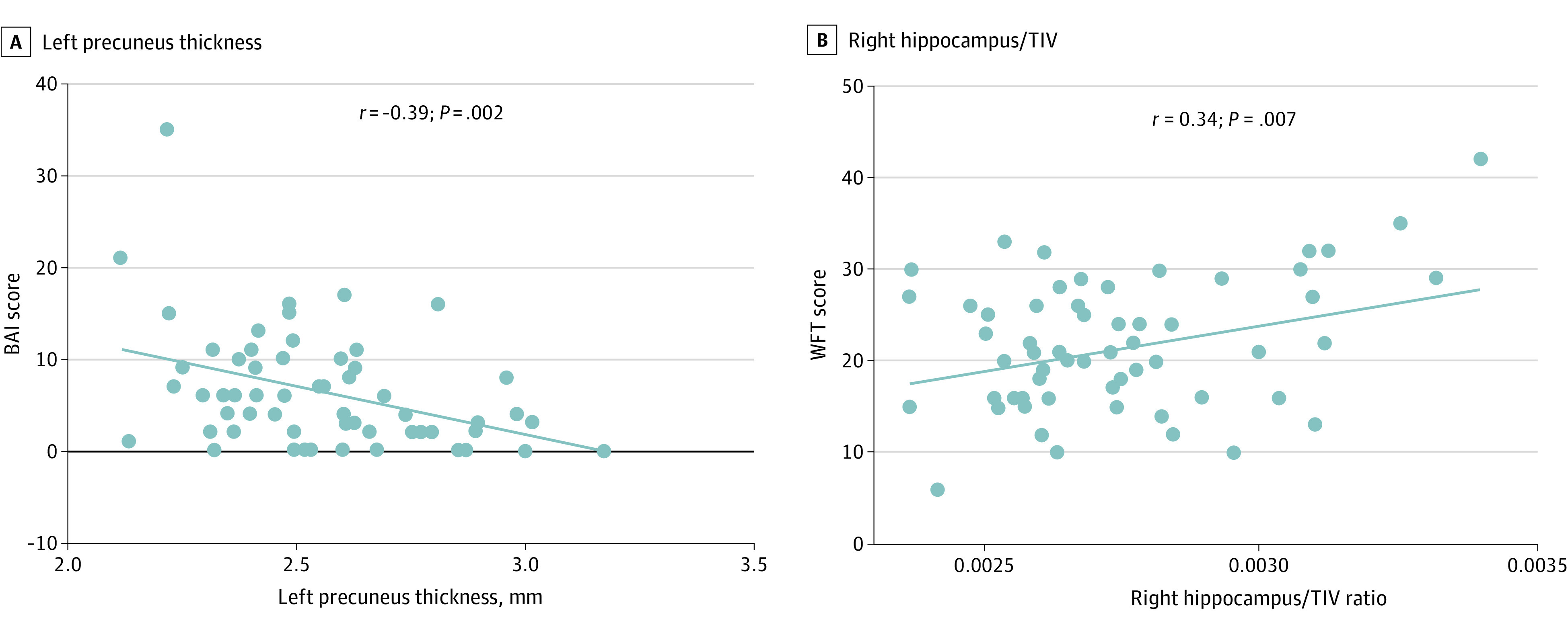
Post-Omicron Correlation Analysis Results The pre-Omicron measurements were obtained before Omicron infection; the post-Omicron measurements, during the acute phase of follow-up (14-29 days). A, The thickness in the left precuneus was negatively correlated with the Beck Anxiety Inventory (BAI) (false discovery rate *P* = .02). B, The difference in the ratio of the right hippocampus to total intracranial volume (TIV) was positively correlated with the Word Fluency Test (WFT) scores.

Compared with pre-Omicron measures, the thicknesses of the left precuneus (mean [SD], 2.7 [0.3] to 2.6 [0.2] mm; *P* < .001), right lateral occipital region (mean [SD], 2.8 [0.2] to 2.7 [0.2] and 2.5 [0.2] to 2.5 [0.2] mm; *P* < .001 for both) (Figure 2A and B), and the ratio of the right hippocampus volume to TIV (mean [SD], 0.003 [0.0003] to 0.003 [0.0002]; *P* = .04) (Figure 2D) were significantly reduced in the post-Omicron follow-up. The febrile group had a significantly reduced sulcus depth of the right inferior parietal region compared with the nonfebrile group (mean [SD], 3.9 [2.3] to 4.8 [1.1]; *P* = .048) (Figure 2C), but there was no significant difference in the ratio of subcutaneous nuclei volumes to the TIV between the 2 groups. Details and post hoc region-of-interest analyses are shown in eTable 2 in Supplement 1. At the post-Omicron follow-up, the *thickness* in the left precuneus was negatively correlated with the BAI (Figure 3A) (*r* = −0.39; *P* = .002; FDR *P* = .02), and the ratio of right hippocampus volume to the TIV was positively correlated with WFT scores (Figure 3B) (*r* = 0.34; *P* = .007). The correlation analyses between the ratio of the right hippocampus volume and the TIV and WFT scores were not significant after FDR. There was no correlation between the sulcus depth and the neuropsychiatric and neurocognitive test scales in the febrile group.

## Discussion

This was the first study, to our knowledge, in which the same participants were examined with MRI before and after an Omicron infection. The main findings were as follows: (1) BAI scores increased and DES scores decreased at the post-Omicron follow-up; (2) neurological symptoms were more common at the post-Omicron follow-up, and fever, myalgia, and cough had improved significantly at the 3-month follow-up; (3) the thicknesses of the left precuneus and right lateral occipital region and the ratio of right hippocampus volume to the TIV were reduced at the post-Omicron follow-up and the sulcus depth was reduced in the right inferior parietal region in the febrile group; and (4) reduced thickness in the left precuneus was correlated with BAI scores and the ratio of right hippocampus volume to the TIV was correlated with WFT scores.

Compared with pre-Omicron measures, we found a significant increase in symptoms of anxiety and a reduced ability to manage negative emotions in participants in the post-Omicron phase. One previous study showed that at least one-third of young adults exhibited depression, anxiety, and posttraumatic stress disorder during the first weeks of the COVID-19 pandemic,^18^ and these symptoms were found to persist for 3 months after recovery.^19,20,21^ A study using the RESE scale to assess mood among patients who recovered from COVID-19^22^ found that positive emotions, anger-irritation, and DES scores were all decreased. Previous findings showed more pronounced neuropsychiatric health problems than those observed herein, which we believe may be related to 2 factors. First, the participants in this study had a different understanding of SARS-CoV-2. Most of the previous studies were conducted at the early stages of the COVID-19 outbreak or in highly exposed populations, such as physicians. Those participants had more fears and worries regarding SARS-CoV-2. However, our study was conducted 3 years after the beginning of the COVID-19 pandemic, and the participants had a clearer understanding of the process of Omicron infection. Second, previous policies required individuals to be isolated after infection, while our participants could be cared for by family and friends during infection. A previous study^18^ found that high levels of isolation were associated with high levels of depression, anxiety, and posttraumatic stress disorder. Therefore, the neuropsychiatric symptoms observed in our study results were milder.

Most studies have focused on comparing the differences in clinical symptoms between the Delta and Omicron variants, and their findings have not been particularly consistent. Reportedly, both variants have similar COVID sequelae in the acute and subacute phases of infection, and the sequelae of Omicron in the chronic phase are milder than those of Delta.^23^ Menni et al^24^ showed that patients infected during the Omicron phase had significantly lower admission rates than those infected during the Delta phase, and the duration of acute symptoms was shorter during the Omicron phase (mean duration of 6.87 days). This finding was consistent with our results; although fever, headache, fatigue, myalgia, cough, and dyspnea were the main symptoms during the post-Omicron follow-up (range of mean [SD] duration, 1.4 [0.6] days for fever to 11.0 [9.5] days for dyspnea), significant improvements in fever, myalgia, and cough were observed at the 3-month follow-up, which is consistent with the view that individuals with an Omicron infection appear to recover more quickly and have milder clinical symptoms than those infected by previous variants.^25,26^

The thickness of the left precuneus and right lateral occipital were reduced during the post-Omicron follow-up compared with pre-Omicron measurements. However, previous follow-up studies^27,28,29^ have shown cortical thickness decreases in areas associated with primary olfactory cortex function during acute, subacute, and 3-month periods after infection with Delta. This may be related to the differences in the mechanism of invasion into the nervous system, neuroinvasiveness, and neurotropism of different variants of SARS-CoV-2^30,31^; therefore, the damaged brain areas and neurotoxic effects may also differ. Because the olfactory epithelium has the highest viral load (sustentacular cells in the olfactory epithelium express both angiotensin-converting enzyme 2 and transmembrane protease serine 2 [TMPRSS2]),^32^ and many patients experience olfactory loss after Delta infection,^33^ most researchers believe that the SARS-CoV-2 Delta variant enters the nervous system via the olfactory nerve and causes a decrease in the volume of gray matter in the relevant brain regions.^28^ Omicron is more likely to enter the nervous system via the terminal nerve pathway, which expresses angiotensin-converting enzyme 2 but not TMPRSS2.^34^ Omicron may enter the body more efficiently by using endosomal host cells rather than through the TMPRSS2-mediated pathway,^35^ so changes in the damaged brain region occur. We found lateralization of the results. The reasons for these lateralized impairments may be different from the mechanism of Omicron damage to the cortex and subcortical nucleus or related to the development of brain lateralization.^36^ More studies may be needed to explore the mechanisms in the future. In addition, the thickness in the left precuneus was negatively correlated with BAI. The precuneus was involved in the manipulation of mental images and internally directing attention from visuospatial images, indicating their unique ability to represent the inner self psychologically. Some studies have confirmed the importance of the precuneus in anxiety neural circuits and psychological interventions for mood and anxiety disorders.^37,38^

The ratio of the right hippocampus volume to the TIV was significantly reduced at the post-Omicron follow-up and was positively correlated with the WFT scores. An animal experiment on human coronavirus OC43 infection showed that in the acute phase of the disease, some infected and uninfected neurons, particularly in the hippocampus, underwent simultaneous apoptosis.^39^ Several studies^40,41^ have shown that neuronal cell apoptosis appears to be an important factor in damage to the human central nervous system with influenza and human immunodeficiency virus infection, potentially revealing the cause of cognitive dysfunction in SARS-CoV-2 infection. A 3-month follow-up study after infection with SARS-CoV-2 showed a reduction in hippocampal cortex thickness,^29^ and another multimodal MRI study found a significant association between gray matter atrophy and cognitive dysfunction.^7^ In addition, the febrile group had reduced sulcus depth of the right inferior parietal compared with the nonfebrile group, and there was no correlation between the sulcus depth and the neuropsychiatric and neurocognitive scale results. This may be related to the fact that the nervous system of a patient with fever experiences more severe hypoxia or inflammatory storms.^29,42^

### Limitations

Our study has some limitations. First, although both the Global Initiative on Sharing Avian Influenza Data report^1^ and the genetic sequencing results of our inpatients indicated that the Omicron variant was dominant in the December 2022 outbreak in China, the participants included in our study were tested only for nucleic acids and did not undergo genetic sequencing. Second, our participants were all men. Third, the symptom data collected at the time of infection were reported based on the participants’ memory, and the number of participants who volunteered to participate in the second clinical symptom collection via the internet was low due to the workload of participation. Fourth, the data on cognitive measures were not collected at the first MRI examination. Fifth, we only conducted 1 follow-up MRI; we will continue to observe the changes in brain structure in the subacute and chronic phases in the future.

## Conclusions

In this cohort study of patients infected with the Omicron variant, the duration of symptoms in multiple systems after infection was short, and fever, myalgia, and cough had obviously improved at the 3-month follow-up. Gray matter thickness and subcortical nuclear volume injury were evident in male patients who recovered from SARS-CoV-2 Omicron infection at the acute-phase follow-up. These findings provide new insights into the emotional and cognitive mechanisms of Omicron invasion into the nervous system and provide an imaging basis for early detection and intervention for neurological sequelae.

## Notes

Supplement 1. **eFigure.** Clinical Symptoms in the Acute Phase and 3-Month Follow-up **eTable 1.** Characteristics of the Febrile and Nonfebrile Groups **eTable 2.** Significant Index Differences Between Pre-Omicron and Post-Omicron Groups and Febrile and Nonfebrile Groups

Supplement 2.
Data Sharing Statement
