# Supplementary material for: Gray Matter Thickness and Subcortical Nuclear Volume in Men After SARS-CoV-2 Omicron Infection
Source: JAMA Netw Open. 2023 Nov 30;6(11):e2345626. doi: 10.1001/jamanetworkopen.2023.45626 (PMC10690469; doi:10.1001/jamanetworkopen.2023.45626)
Supplement: Supplement 1. — eFigure. Clinical Symptoms in the Acute Phase and 3-Month Follow-up eTable 1. Characteristics of the Febrile and Nonfebrile Groups eTable 2. Significant Index Differences Between Pre-Omicron and Post-Omicron Groups and Febrile and Nonfebrile Groups [file jamanetwopen-e2345626-s001.pdf]

# Supplementary Online Content

Du Y, Zhao W, Huang S, et al. Gray Matter Thickness and Subcortical Nuclear Volume in Men After SARS-CoV-2 Omicron Infection. *JAMA Netw Open*. 2023;6(11):e2345626. doi:10.1001/jamanetworkopen.2023.45626

**eFigure.** Clinical Symptoms in the Acute Phase and 3-Month Follow-Up
**eTable 1.** Characteristics of the Febrile and Nonfebrile Groups
**eTable 2.** Significant Index Differences Between Pre-Omicron and Post-Omicron Groups and Febrile and Nonfebrile Groups

This supplementary material has been provided by the authors to give readers additional information about their work.

© 2023 Du Y et al. *JAMA Network Open*.

**eFigure.** Clinical Symptoms in the Acute Phase and 3-Month Follow-Up

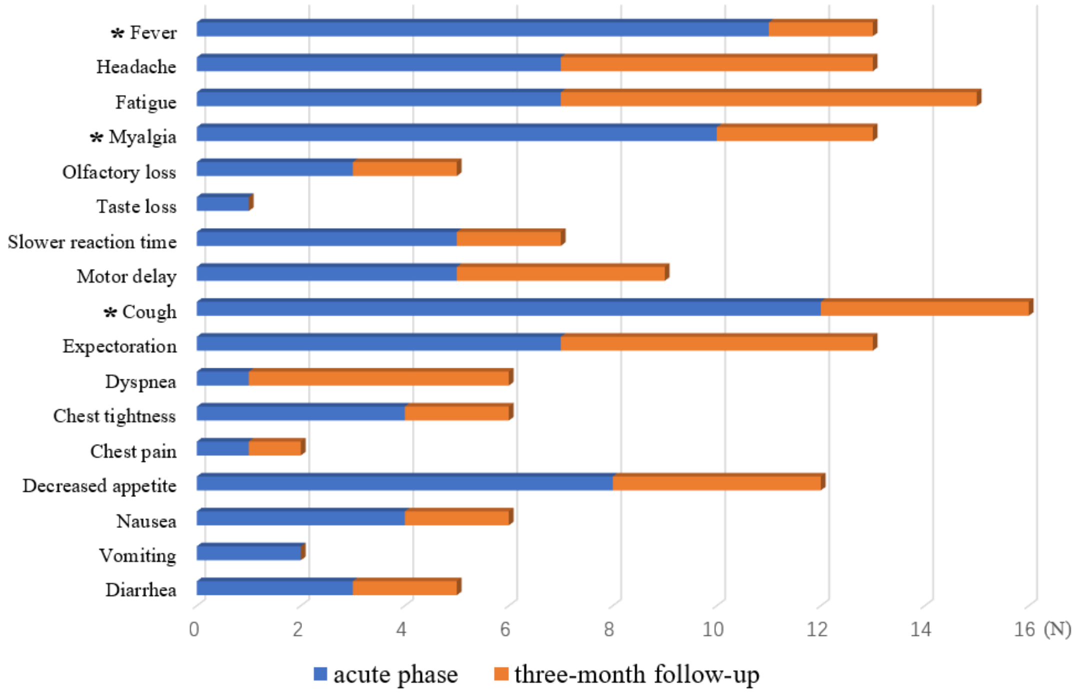


Compared with the acute phase, fever, myalgia, and cough improved significantly in the 17 individuals who were followed up after three-month follow-up. * McNemar's test. N: number of subjects. *P* values less than 0.05 indicate statistical significance.

© 2023 Du Y et al. *JAMA Network Open*.

**eTable 1.** Characteristics of the Febrile and Nonfebrile Groups

| **Characteristics** | **Febrile** [#] **(N=36)** | **Nonfebrile (N=25)** | **t /$\chi^2$ /Z** | ***P*** |
|---|---|---|---|---|
| Age (years)[a] | 40.35±9.82 | 47.48±8.32 | -2.832 | **0.006**[*] |
| Gender (male/female)[c] | 36/0 | 25/0 | - | - |
| Education (years)[b] | 16.00(15.50, 16.00) | 16.00(15.00, 16.00) | -0.192 | 0.847 |
| BMI ($kg/m^2$)[b] | 24.69(23.62, 27.27) | 26.64(22.02, 28.01) | -0.568 | 0.570 |
| Nicotine use (N, %)[c] | 16, 44.44% | 18, 72% | 4.541 | **0.033**[*] |
| Alcohol use (N, %)[c] | 19, 52.78% | 17, 68% | 1.413 | 0.234 |
| Hypertension (N, %)[c] | 3, 8.33% | 6, 24% | 1.768 | 0.184 |
| Diabetes Mellitus (N, %)[c] | 3, 8.33% | 3, 12% | 0.001 | 0.971 |
| Handedness | 36R | 25R | - | - |
| Time interval between Omicron infection and MR examination[a] | 22.09±5.43 | 20.95±5.15 | 0.693 | 0.491 |
| **Vaccination Status**[c] | | | | |
| Single or double vaccinated | 16 | 13 | 0.411 | 0.814 |
| Booster vaccinated | 11 | 6 | | |
| Missing | 9 | 6 | | |
| BDI[b] | 1.00(0.00, 2.50) | 0.50(0.00, 2.25) | -1.236 | 0.217 |
| BAI[b] | 4.00(2.00, 9.50) | 5.00(1.75, 11.25) | -0.597 | 0.550 |
| ISI[b] | 5.00(2.00, 7.00) | 3.50(0.00, 7.50) | -1.118 | 0.264 |
| **RESE** | | | | |
| POS[b] | 13.00(12.00, 15.00) | 12.00(12.00, 15.00) | -0.144 | 0.885 |
| ANG[b] | 16.00(13.50, 18.50) | 14.00(11.75, 17.25) | -0.766 | 0.444 |
| DES[b] | 16.00(15.00, 20.00) | 16.00(15.00, 18.00) | -0.816 | 0.415 |

© 2023 Du Y et al. *JAMA Network Open*.

| | | | | |
|---|---|---|---|---|
| **Neurocognitive tests** | | | | |
| LM-A[a] | 10.12±3.89 | 10.32±3.91 | -0.143 | 0.887 |
| LM-B[a] | 7.79±4.42 | 9.00±3.44 | -0.826 | 0.412 |
| DSST[a] | 81.76±18.46 | 73.91±23.71 | 1.683 | 0.098 |
| Knowledge subscale of Wechsler Intelligence scale[a] | 23.67±3.34 | 22.64±4.68 | 0.654 | 0.516 |
| FDS[b] | 14.00(11.50, 14.00) | 14.00(12.00, 14.00) | -1.395 | 0.163 |
| BDS[b] | 9.00(7.00, 14.00) | 8.00(6.00, 12.00) | -0.720 | 0.472 |
| WFT[a] | 22.36±6.61 | 20.92±7.45 | 0.820 | 0.415 |

[a] Two-sample t-test. [b] Two-sample Wilcoxon-Mann-Whitney U test. [c] Chi-square test. [#] Fever was defined as an axillary temperature above 38.5°C. N: number of subjects. BMI: body mass index. R: right. BDI: Beck Depression Inventory. BAI: Beck Anxiety Inventory. ISI: insomnia severity index. RESE: regulatory emotional self-efficacy scale. POS: expressing positive emotions. ANG: managing anger-irritation. DES: managing despondency-distress. LM: logical memory task. DSST: digital symbol substitution test. FDS: forward digit span. BDS: backward digit span. WFT: Word fluency test. * *P* values less than 0.05 indicate statistical significance.

© 2023 Du Y et al. *JAMA Network Open*.

**eTable 2.** Significant Index Differences Between Pre-Omicron and Post-Omicron Groups and Febrile and Nonfebrile Groups

| **Index** | **Brain regions** | **Side (R/L)** | **Size** | **MNI coordinate** | | | **Values** | | ***P*** |
|---|---|---|---|---|---|---|---|---|---|
| | | | | **X** | **Y** | **Z** | | | |
| **Pre-Omicron vs Post-Omicron (61 vs 61)** | | | | | | | | | |
| Thickness(mm) | precuneus | L | 158.72 | -6.2 | -62.2 | 30.6 | 2.65±0.26 | 2.56±0.24 | <0.001 |
| | lateral occipital | R | 581.49 | -23.8 | -95.1 | 14.8 | 2.80±0.18 | 2.73±0.19 | <0.001 |
| | lateral occipital | R | 418.45 | -7.8 | -101.2 | 5.5 | 2.54±0.17 | 2.49±0.18 | <0.001 |
| Subcortical volumes | hippocampus/TIV | R | - | - | - | - | 0.003±0.0003 | 0.003±0.0002 | 0.044 |
| **Febrile vs - Nonfebrile (36 vs 25)** | | | | | | | | | |
| Sulcus depth | inferior parietal | R | 179.76 | 34.9 | -74.3 | 27.5 | 3.87±2.27 | 4.77±1.07 | 0.048 |

R: right. L: left. MNI: Montreal Neurological Institute. Pre-Omicron: before infection with Omicron. Post-Omicron: acute phase of Omicron infection. TIV: total intracranial volume. hippocampus/TIV: ratio of the hippocampus volume to the TIV.

© 2023 Du Y et al. *JAMA Network Open*.
