# Supplementary material for: Gray Matter Thickness and Subcortical Nuclear Volume in Men After SARS-CoV-2 Omicron Infection
Source: JAMA Netw Open. 2023 Nov 30;6(11):e2345626. doi: 10.1001/jamanetworkopen.2023.45626 (PMC10690469; doi:10.1001/jamanetworkopen.2023.45626)
Supplement: Supplement 2. — Data Sharing Statement [file jamanetwopen-e2345626-s002.pdf]

Data Sharing Statement

Du. Gray Matter Thickness and Subcortical Nuclear Volume in Men After SARS-CoV-2 Omicron Infection. *JAMA Netw Open*. Published November 30, 2023. doi:10.1001/jamanetworkopen.2023.45626

Data
Data available: Yes
Data types: Data (not involving human participants)
How to access data: After publication, the data will be made available to other researchers on reasonable request to the corresponding author.
When available: With publication

Supporting Documents
Document types: Informed consent form
How to access documents: After publication, the data will be made available to other researchers on reasonable request to the corresponding author.
When available: With publication

Additional Information
Who can access the data: researchers whose proposed use of the data has been approved
Types of analyses: for any purpose
Mechanisms of data availability: with a signed data access agreement
